# Examining cost methodologies in survey-based observational studies in stroke: A protocol for a scoping review

**DOI:** 10.12688/hrbopenres.14213.2

**Published:** 2025-11-10

**Authors:** Tumeliwa Mphepo, Sreemeena Satyanarayana, Gintare Valentelyte, Jan Sorensen

**Affiliations:** 1 Healthcare Outcomes Research Centre (HORC), School of Population Health, Royal College of Surgeons in Ireland 26 York Street Campus, Dublin, Leinster, D02 YN77, Ireland; 2 Converge: Centre for Chronic Disease and Population Health Research, School of Population Health,, Royal College of Surgeons in Ireland 26 York Street Campus, Dublin, Leinster, D02 YN77, Ireland

**Keywords:** cost analyses, cost methodology, cost of illness studies, observational studies, scoping review, stroke, survey

## Abstract

**Background:**

Stroke poses a significant burden globally, making economic evaluations important. However, differences in cost methodologies and data sources challenge the interpretability and comparability across studies. Additionally, stroke presents challenges related to memory recall, affecting the reliability of patient-reported outcomes. While previous reviews have identified variations in cost methodologies within stroke research, there is a lack of literature specifically examining the cost methods used in survey-based observational studies, particularly given the distinct complexities associated with stroke. This protocol outlines a scoping review to identify and examine the cost methodologies employed in stroke research, focusing on data collection approaches, measurement and valuation of cost components, and sources of cost information. The review will also document the limitations highlighted in the identified studies regarding the cost methods and their recommendations for future studies.

**Methods:**

The JBI methodology will be used to develop this review. Articles will be sourced from Embase, PubMed, and Web of Science. The Population, Concept, and Context framework will guide the inclusion and exclusion criteria. Observational studies involving stroke patients or survivors aged 18 years and older that used surveys for data collection and incorporated a cost analysis component, and published in English within the last 20 years across all geographical regions will be considered. Articles will be entered into Covidence, where two reviewers will independently screen and extract/check data. A data collection template will guide the data extraction process for analysis and narrative synthesis. The Preferred Reporting Items for Systematic Reviews and Meta-analysis extension for Scoping Reviews will guide the reporting.

**Conclusion:**

This scoping review will examine the cost methodologies used in survey-based observational studies in stroke research. The findings may inform the cost methodologies employed in future observational studies in stroke using surveys as a data collection tool.

## Introduction

Non-communicable diseases (NCDs) are a global public health concern, with cardiovascular disease, cancer, chronic respiratory disease and diabetes being the major contributors ^1^. From 1990 to 2019, NCDs accounted for the highest absolute increase in Disability-Adjusted Life Years (DALYs) ^2^. In 2021, seven of the ten leading causes of death worldwide were attributed to NCDs, with ischemic heart disease at the top and stroke ranking third ^3^. That same year, stroke accounted for the greatest loss in global DALYs among diseases affecting the nervous system and was the leading cause of DALY loss in 19 of the 21 Global Burden of Disease (GBD) regions ^4^.

The burden of stroke is apparent in both the increased mortality and morbidity, with approximately 50% of survivors remaining chronically disabled ^5^. Although advances in stroke management have significantly reduced age-standardised incidence, mortality and DALYs, and have increased life expectancy by shifting the burden to older populations ^6, 7^, several studies have reported an increase in the incidence and prevalence of stroke among individuals aged 65 years or younger ^8– 11^. The longer duration spent living with stroke translates to an increased economic burden ^12– 14^. Therefore, conducting costing studies in stroke is important for accurately assessing the burden of disease and prioritising interventions ^15^.

Cost of Illness (COI) studies estimate various costs, including inpatient, outpatient, and other types of care, indirect costs stemming from work absenteeism, presenteeism, temporary or lifelong disabilities, death, and intangible costs ^16^. While this information is important for informing evidence-based decisions, understanding the methodologies used to collect these costs is equally important, as the methods directly impact the accuracy of the estimates. However, despite providing detailed information on economic losses, often, COI studies do not consider additional outcomes such as the associated disutility linked to having the disease, making it difficult to draw meaningful comparisons with other disease areas in terms of cost, benefits, or consequences ^17, 18^. Conversely, cost analyses focused on Healthcare Expenditure (HE) primarily capture costs related to healthcare services, ignoring the broader economic impacts on patients, their households, and society as a whole ^17^.

Previous systematic reviews on the costs associated with stroke have highlighted significant differences in the methodologies used across studies, with many not clearly defining the cost estimation approaches adopted ^16^. These reviews primarily focused on whether a study used a top-down or bottom-up approach, rather than examining whether cost data were obtained directly from stroke patients, their caregivers, or medical professionals. Similarly, another systematic review on the cost of post-stroke care reported that cost data sources included hospital records, insurance data, local or national registries, and questionnaires, but did not specify the source of this information (primary source) ^19^. This lack of clarity limits interpretability, hinders meaningful comparisons across studies and may introduce bias into economic evaluations, as cost estimates derived from patients may differ from those reported by caregivers, even under the same societal perspective. Therefore, challenges in comparing the cost of stroke across different studies persist due to variations in methodologies employed and data sources.

We are motivated to conduct this study after our previous experience conducting a cross-sectional survey on “ *the costs associated with accessing healthcare related to stroke diagnosis in Ireland*”, which revealed challenges in data collection. There was a low response rate, as many of the invited individuals with strokes did not participate in the survey. Among those who participated, the response rates declined with cost-related questions. In one group (n=38), only 24 (63%) answered the first cost question, and only 18 (47%) answered the final question. In another group (n=52), only 29 (56%) completed the cost section. Additionally, some respondents reported difficulty recalling costs, which highlighted potential recall bias in self-reported healthcare expenditures. Studies have reported that cognitive and memory impairments are common following a stroke ^20^, which may affect patients’ ability to accurately recall healthcare-related expenses. This raises important considerations about the reliability of patient-reported cost data in stroke studies. However, if such data are questioned and possibly replaced by other sources, it would be challenging to capture intangible costs such as pain and emotional distress that are difficult to value without actual personal experience of the disease.

This protocol outlines the approach of a scoping review to document how previous studies of costs related to stroke have been conducted, and especially consider whether and how the authors have addressed the cognitive consequences of respondents' stroke on their survey results.

Specifically, the scoping review seeks to:

1. Identify the cost estimation approaches used (e.g., activity-based costing, micro-costing, macro-costing) and the cost perspectives adopted (e.g., healthcare, societal)
2. Identify the cost components measured and how these were valued
3. Assess the methodology and transparency of the primary source of the cost data (i.e., who responded to the survey)
4. Assess the length of the period respondents had to provide recalled cost data
5. Explore challenges and limitations encountered in cost and estimation and summarise potential solutions or recommendations for improving the quality of future cost studies in this area
6. Explore if and how the study design has accounted for potential cognitive decline in respondents.

The findings from this review will help researchers, policymakers, and healthcare providers to identify the most appropriate approach to study stroke costs. It will outline how costing methodologies may be standardised for observational stroke studies to ensure accuracy of cost estimates and facilitate comparison across studies.

## Protocol

### Design

We will conduct a methodological scoping review of survey-based observational studies exploring the costing of stroke. This scoping review will be conducted following the JBI methodology for conducting scoping reviews ^21^, which builds on the stages of conducting scoping reviews as proposed by Arksey and O’Malley ^22^. The Preferred Reporting Items for Systematic Reviews and Meta-analysis extension for Scoping Reviews (PRISMA-Scr) will guide the reporting ^23^.

We acknowledge that currently, there is no PRISMA-ScR protocol checklist to guide the reporting of scoping review protocols. While the Preferred Reporting Items for Systematic Review and Meta-Analysis Protocols (PRISMA-P) checklist exists to support the reporting of systematic review protocols ^24, 25^, it is not applicable to our study due to the methodological differences between systematic and scoping reviews. Therefore, we used the PRISMA-ScR checklist and reported only the items that are relevant and appropriate for scoping review protocols.

### Stage 1: Identifying the research question

The PCC (Population, Concept, Context) framework has been applied to formulate the research question and to define the inclusion and exclusion criteria.

### Review question

What methodologies have been used and applied in survey-based observational studies to estimate costs related to stroke?

### Population

The study will include published academic studies of adult stroke patients or survivors (aged 18 years and older). The review will include different types of strokes, provided the population is within the specified age range.

### Concept

The concept is cost analysis methods. Studies that conducted a cost analysis using data from a survey questionnaire will be included. The cost analysis may be conducted in the context of a cost-of-illness study or an economic evaluation, such as a cost-utility analysis, cost-effectiveness analysis, cost-consequence analysis, cost-minimisation analysis, or cost-benefit analysis.

### Context

The review will include articles from across the world's geographical regions, regardless of differences in socio-economic status across countries and regions. The review will focus on studies published in the last 20 years (2005–2025). Twenty years has been chosen to ensure we gather the latest and most relevant information, which could also be useful for future studies. A summary of the inclusion and exclusion criteria is presented in Table 1.

**Table 1.** Inclusion and exclusion criteria. Table 1 summarises the eligibility criteria for studies to be included in the review using the Population, Concept and Context framework.

Category_Inclusion Criteria	Exclusion Criteria	
Population	• Studies conducted on stroke patients/survivors • All types of strokes, including haemorrhagic, ischaemic, and Transient Ischaemic Attack (TIA) • Adult stroke patients/survivors (i.e., aged ≥18 years)	• Studies conducted not on stroke patients/survivors • Paediatric stroke patients/survivors (i.e., aged <18 years)
Concept	Cost analysis methods conducted using data from survey-based observational studies. The review will include studies that conducted: • Cost of illness studies • Other health economic evaluations, including (cost-utility analysis, cost-effectiveness analysis, cost-consequence analysis, cost-minimisation analysis, or cost-benefit analysis) using primary data	Cost analysis methods conducted using data from non-survey-based observational studies, including: • Cost-of-illness studies using cost data from secondary, administrative, or simulation sources • Other health economic evaluations, including (cost-utility analysis, cost-effectiveness analysis, cost-consequence analysis, cost-minimisation analysis, or cost-benefit analysis), using cost data from secondary, administrative or simulation sources
Context	• Studies involving human participants • Studies across the world • Studies published in the English language • Studies published since the year 2005 (≥2005)	• Studies conducted exclusively on animals • Studies published in languages other than English • Studies published before the year 2005 (<2005)
Study design	• Observational studies • Prospective and retrospective cohort studies • Case-control studies • Cross-sectional studies • Cost of illness studies • Time series studies	• Qualitative studies • Systematic literature reviews, scoping reviews and other types of reviews • Opinion papers • Commentaries, editorials, letters, conference abstracts • Protocols • Randomised Controlled Trials • Non-Randomised Controlled Trials • Post-trial follow-up RCTs or non-RCTs with survey-based cost data • Health economic evaluations using model-based economic evaluations or secondary data sources

### Stage 2: Identifying relevant studies

This scoping review will consider observational studies, including analytical observational studies such as prospective and retrospective cohort studies, case-control studies, analytical cross-sectional studies, cost-of-illness studies, and time-series studies. Given that the review will focus on observational studies, all experimental and quasi-experimental designs, including randomised and non-randomised controlled trials, will be excluded.

All qualitative studies on stroke will also be excluded. In addition, cost studies that use a model-based economic evaluation or secondary data sources, as well as systematic literature reviews, other types of reviews, opinion papers, commentaries, and protocols, will be excluded.

This scoping review will be conducted using two key search terms: “stroke” and “cost”. One reviewer (TM), in collaboration with the RCSI information specialist, will develop a pre-defined search strategy that will be used to conduct a comprehensive search on electronic databases ( Embase, PubMed, and Web of Science). The first search will be conducted in PubMed to identify the Medical Subject Headings (MeSH) terms, index terms and subject headings used in the titles and abstracts of relevant articles. The identified words and terms will be adapted for each included database and/or information source. The review will focus on studies published in English, as the review team lacks expertise in translating studies from other languages. The full search strategy with MeSH terms and keywords is presented in Table 2.

**Table 2.** Search strategies across all databases. Table 2 presents coined search strategies for Embase, PubMed, and Web of Science. The table also includes a year-of-publication filter that will be applied to the search for relevant studies.

Embase Query	
***Stroke keywords***	
1	'cerebrovascular accident'/exp OR 'stroke':ti,ab OR 'apoplexy':ti,ab OR 'cva':ti,ab OR 'cerebral stroke*':ti,ab OR 'cerebrovascular accident, acute':ti,ab OR 'cerebrovascular apoplexy':ti,ab OR 'cerebrovascular stroke*':ti,ab OR 'stroke, acute':ti,ab OR 'vascular accident, brain':ti,ab OR 'cerebrovascular disorder*':ti,ab OR 'intracranial arteriosclerosis':ti,ab OR 'intracranial embolism and thrombosis':ti,ab OR 'cerebrovascular accident*':ti,ab
***Cost keywords***	
2	'cost benefit analysis'/exp OR 'cost effectiveness analysis'/exp OR 'cost utility analysis'/exp OR 'cost minimization analysis'/exp OR 'economic evaluation'/exp OR 'cost'/exp OR 'cost':ti,ab OR 'cost analys*':ti,ab OR 'cost benefit analys*':ti,ab OR 'cost-effectiveness':ti,ab OR 'cost effectiveness analys*':ti,ab OR 'cost-effectiveness analys*':ti,ab OR 'cost utility analys*':ti,ab OR 'cost-utility analys*':ti,ab OR 'cost minimi* analys*':ti,ab OR 'cost-minimi* analys*':ti,ab OR 'cost consequence analys*':ti,ab OR 'cost-conseque* analys*':ti,ab OR 'economic*':ti,ab OR 'economic evaluation*':ti,ab OR cost*:ti,ab OR 'budget impact analys*':ti,ab OR 'budget-impact analys*':ti,ab
3	1 AND 2
PubMed Query	
***Stroke keywords***	
1	"Stroke"[Mesh] OR "Stroke*"[Title/Abstract] OR "Apoplexy"[Title/Abstract] OR "CVA"[Title/Abstract] OR "Cerebrovascular Accident*"[Title/Abstract] OR "Cerebral Stroke"[Title/Abstract] OR "Cerebrovascular Accident*"[Title/Abstract] OR "Cerebrovascular Accident, Acute"[Title/Abstract] OR "Cerebrovascular Apoplexy"[Title/Abstract] OR "Cerebrovascular Stroke*"[Title/Abstract] OR "Stroke, Acute"[Title/Abstract] OR "Vascular Accident, Brain"[Title/Abstract] OR "Cerebrovascular Disorder*"[Title/Abstract] OR "Intracranial Arteriosclerosis"[Title/Abstract] OR "Intracranial Embolism and Thrombosis"[Title/Abstract] OR "Hemorrhagic Stroke*"[Title/Abstract] OR "Haemorrhagic Stroke*"[Title/Abstract] OR "Embolic Stroke*"[Title/Abstract] OR "Thrombotic Stroke*"[Title/Abstract] OR "cerebrovascular insult*"[Title/Abstract] OR "CVI"[Title/Abstract] OR "Cerebrovascular lesion*"[Title/Abstract] OR "CVL"[Title/Abstract] OR "brain attack*"[Title/Abstract]
***Cost keywords***	
2	"Cost*"[Mesh] OR "Costs and Cost Analysis"[Mesh] OR "Economics"[Mesh] OR "Cost-Benefit Analysis"[Mesh] OR "Cost-Effectiveness Analysis"[Mesh] OR "cost"[Title/Abstract] OR "cost analys*"[Title/Abstract] OR "cost benefit analys*"[Title/Abstract] OR "cost-effectiveness"[Title/Abstract] OR "cost effectiveness analys*"[Title/Abstract] OR "cost-effectiveness analys*"[Title/Abstract] OR "cost utility analys*"[Title/Abstract] OR "cost-utility analys*"[Title/Abstract] OR "cost minimi* analys*"[Title/Abstract] OR "cost-minimi* analys*"[Title/Abstract] OR "cost consequence analys*"[Title/Abstract] OR "cost-conseque* analys*"[Title/Abstract] OR "economic*"[Title/Abstract] OR "economic evaluation*"[Title/Abstract] OR "cost*"[Title/Abstract] OR "budget impact analys*"[Title/Abstract] OR "budget-impact analys*"[Title/Abstract]
3	1 AND 2
Web of Science Query	
***Stroke keywords***	
1	TS=( "stroke" OR "apoplexy" OR "cva" OR "cerebral stroke*" OR "cerebrovascular accident, acute" OR "cerebrovascular apoplexy" OR "cerebrovascular stroke*" OR "stroke, acute" OR "vascular accident, brain" OR "cerebrovascular disorder*" OR "intracranial arteriosclerosis" OR "intracranial embolism and thrombosis" OR "cerebrovascular accident*" )
***Cost keywords***	
2	TS=("cost" OR "cost analys*" OR "cost benefit analys*" OR "cost-effectiveness" OR "cost effectiveness analys*" OR "cost-effectiveness analys*" OR "cost utility analys*" OR "cost-utility analys*" OR "cost minimi* analys*" OR "cost-minimi* analys*" OR "cost consequence analys*" OR "cost-conseque* analys*" OR "economic*" OR "economic evaluation*" OR "cost*" OR "budget impact analys*" OR "budget-impact analys*"
3	1 AND 2
***Applied filters across all databases***	
Publication dates 2005/1/1 - 2025/12/31	

### Stage 3: Study selection

After the search, all study articles will be imported into EndNote version 21 to convert them into a format compatible with Covidence. Articles will then be collated and uploaded to Covidence, where duplicates will be removed. The primary reviewer (TM) will remove articles that are clearly irrelevant. Then, two reviewers (TM and SS) will independently perform title and abstract screening against the inclusion and exclusion criteria for the review. The articles included after screening will be put through a full-text review. The full-text review will assess the included articles in detail against the inclusion criteria. The reasons for excluding articles at this stage will be recorded and reported in the review. Any disagreements between the reviewers at each stage of the selection process will be resolved through discussion and by decision of an additional reviewer (GV), and additional discrepancies will be resolved with input from another reviewer (JS). The results of the search and the study inclusion process will be reported in full in the final scoping review and presented in a PRISMA flow diagram.

### Stage 4: Charting the data

Data from the articles will be extracted by one reviewer (TM) and checked by the second reviewer (SS) for accuracy. A data extraction tool will be developed by the reviewers and published in Covidence. Two independent reviewers (TM and SS) will pilot the data extraction tool with 10% of all identified articles to identify any flaws that require modification. The draft data extraction tool will also be modified and revised as necessary during the process of extracting data from each included article. Modifications will be detailed in the scoping review. Some key information that will be extracted consists of the following:

1. Study identification: author, year of publication, country, healthcare setting, aim of the study
2. Survey design: cross-sectional or cohort, population inclusion/exclusion criteria, survey mode (telephone, online, face-to-face), sampling strategy (probability, convenience), sample size, survey instrument used (adapted, newly developed), pre-testing conducted or not, who completed the questionnaire, cost recall period, respondent incentive given or not
3. Costing methods: perspective adopted (healthcare, societal, patient), costing approach (activity-based, micro-costing, gross costing), resource use (direct, indirect, intangible), unit cost sources (national database, self-reported), time horizon for cost reported
4. Data analysis: statistical methods used, handling of missing data, costs reported as (mean, median, how outliers were handled)
5. Limitations and recommendations: authors’ limitations or challenges regarding cost methodology or survey, and authors’ recommendations given

### Stage 5: Collating, summarising and reporting the results

This review will be analysed descriptively. We will conduct simple frequency and counts of specific variables in relation to the review’s objective. Data will be presented in tables, graphs, and charts. A narrative summary describing how the results relate to the review’s objectives and questions will follow the tabulated, graphical, or charted results.

## Dissemination

The findings of the scoping review will be shared in relevant meetings, published in a peer-reviewed, open-access journal, presented at national and international conferences, and disseminated to other researchers and stakeholders in stroke research who may find this information relevant.

## Study status

The study protocol has been registered on the Open Science Framework:

https://doi.org/10.17605/OSF.IO/S8P9A

## Tracked and dated amendments

Any amendments to this protocol, including the dates of the amendments and justifications, will be documented and presented in a table in the scoping review publication.

## Conclusions

Given the cognitive challenges associated with stroke, it is important to develop a better understanding of the most effective approach to carry out costing in stroke.

## Ethical considerations

Ethical approval and consent were not required.

## Data Availability

No data are associated with this article. Zenodo PRISMA-Scr checklist for “Examining cost methodologies used in survey-based observational studies in stroke: a protocol for a scoping review”. https://doi.org/10.5281/zenodo.15976529 ^26^. Data are available under the terms of the Creative Commons Attribution 4.0 International license (CC-BY-4.0).
