# Supplementary material for: Examining cost methodologies in survey-based observational studies in stroke: A protocol for a scoping review
Source: HRB Open Res. 2025 Nov 10;8:84. Originally published 2025 Jul 28. [Version 2] doi: 10.12688/hrbopenres.14213.2 (PMC12828259; doi:10.12688/hrbopenres.14213.2)
Supplement: Peer review history

# Reviewer report: Reviewer response for version 2

**DOI:** 10.21956/hrbopenres.15712.r52417

**Published:** 2026-01-22

**Authors:** Yuyun Yuniar

**Affiliations:** 1 National Research and Innovation Agency, Indonesia, Sydney, Indonesia

Introduction: a top-down or bottom-up approach

 Please define both terms

 The entire paragraph would benefit from **revision **for better understanding and clarity for the readers.

 Statement no. 2: Identify the cost components measured and how these were valued

 State explicitly whether you classified **direct**, **indirect**, and **opportunity** costs as cost components here

 Protocol

 Suggest renaming **“Protocol”** to **“Methods”**

 Your statement: We acknowledge that currently, there is no PRISMA-ScR protocol checklist…

 Please **check the publication from Tricco (2018)**. There **is** a PRISMA extension for scoping reviews ( **PRISMA‑ScR**) with a **checklist and explanation** (Tricco et al., 2018) (Ref 1). If this aligns with your study, please **update and correct** the statement.

 Based on an editorial by Moher (2015) (Ref 2) a scoping review is also a species of the systematic reviews family. If you agree with the statement, you need to revise the design section explanation to accommodate this issue. Check the publication.

 Population: …survivors

 Define **“survivors.”**

 Context: 20 years

 I share the previous reviewer’s concern.

 Please provide a **substantive rationale** for the **20‑year** timeframe instead of an assumption. Why not 15 or 10 years and consider a **broader range,** including all years before 2005?

 Stage 2: Identifying relevant studies

 I understand from the statement that only primary will be used since the secondary data sources will be excluded. Clarify in the inclusion criteria if my understanding is correct.

 please **provide secondary data explanation **with examples in brackets (e.g., hospital medical records, …)

 Stage 3: Study selection

 The primary reviewer (TM) will remove articles that are clearly irrelevant.

 How “removing clearly irrelevant article” differs from formal **title/abstract screening**

 How will you do the removal?

 Are you planning to conduct a critical appraisal of the sources? Provide the explanation of whether an appraisal will or will not be done.

Is the study design appropriate for the research question?

Yes

Is the rationale for, and objectives of, the study clearly described?

Yes

Are sufficient details of the methods provided to allow replication by others?

Partly

Are the datasets clearly presented in a useable and accessible format?

Not applicable

Reviewer Expertise:

Social pharmacy, pharmaceutical policy

I confirm that I have read this submission and believe that I have an appropriate level of expertise to confirm that it is of an acceptable scientific standard.

## References

[1] : PRISMA Extension for Scoping Reviews (PRISMA-ScR): Checklist and Explanation. *Annals of Internal Medicine* .2018;169(7) : 10.7326/M18-0850 467-473 10.7326/M18-0850 30178033

[2] : All in the Family: systematic reviews, rapid reviews, scoping reviews, realist reviews, and more. *Systematic Reviews* .2015;4(1) : 10.1186/s13643-015-0163-7 10.1186/s13643-015-0163-7 26693720 PMC4688988

# Reviewer report: Reviewer response for version 2

**DOI:** 10.21956/hrbopenres.15712.r51318

**Published:** 2025-11-25

**Authors:** John Grosser

**Affiliations:** 1 Universität Bielefeld, Bielefeld, Germany

Thank you for your prommpt responde to my comments. I have no additional reservations concerning your manuscript. Good luck in conducting your review!

Is the study design appropriate for the research question?

Yes

Is the rationale for, and objectives of, the study clearly described?

Yes

Are sufficient details of the methods provided to allow replication by others?

Partly

Are the datasets clearly presented in a useable and accessible format?

Not applicable

Reviewer Expertise:

Relevant areas of expertise: Stroke, Scoping Reviews

I confirm that I have read this submission and believe that I have an appropriate level of expertise to confirm that it is of an acceptable scientific standard.

# Reviewer report: Reviewer response for version 1

**DOI:** 10.21956/hrbopenres.15631.r49445

**Published:** 2025-09-24

**Authors:** Erik Buskens, Chi Nguyen

**Affiliations:** 1 University Medical Center Groningen, Groningen, The Netherlands; 2 epidemiology university medical center Groningen, University of Groningen (Ringgold ID: 3647), Groningen, The Netherlands

**Examining cost methodologies in survey-based observational studies in stroke: A protocol for a scoping review**

**Strengths: **The protocol addresses an important gap in stroke health economics research. The focus on survey-based observational studies could generate valuable insights for improving cost methodology in future RWD based studies. The manuscript uses JBI methodology and PRISMA-ScR standards to strengthen transparency and reproducibility.

**Areas for Improvement:**

 Could you clarify why your inclusion criteria specify stroke patients aged 19 years and above, while many studies define adults as ≥18 years? You mention that 19 years is the cut-off in PubMed, but since you are also searching Embase and Web of Science, do you think this filter could exclude relevant studies?

 The decision to restrict to English-language publications may introduce language and/or region bias. Economic studies typically pertain to a local or national setting whereas studies on clinical outcomes may be more or less generic. How do you plan to address or acknowledge this limitation in your review? You apply English-language filter in your search. I would suggest keep all languages. Where possible, you may compare results based on English and non-English publication to point out differences (at least based on abstract information).

 Your focus is on survey-based observational studies. Will you also consider cost data collected via questionnaires in long-term follow-up phases of clinical trials? These might be equally informative when trying to establish long-term costs after achieving a short-term clinical treatment effect. Your protocol excludes experimental and quasi-experimental designs (including randomized and non-randomized controlled trials). It may be worth clarifying whether post-trial follow-up RCTs with survey-based cost data will be included or excluded.

 The inclusion of perspective, costing approach, and resource use categories is excellent. Will you also extract details on time horizon (e.g., costs reported over 3 months, 1 year, or longer)? This factor can influence cost estimates and would be valuable to capture.

Is the study design appropriate for the research question?

Partly

Is the rationale for, and objectives of, the study clearly described?

Yes

Are sufficient details of the methods provided to allow replication by others?

Yes

Are the datasets clearly presented in a useable and accessible format?

Not applicable

Reviewer Expertise:

clinical epidemiology, HTA and public health

We confirm that we have read this submission and believe that we have an appropriate level of expertise to confirm that it is of an acceptable scientific standard, however we have significant reservations, as outlined above.

# Response

**Published:** 2025-10-23

**Authors:** Tumeliwa Mphepo

**Affiliations:** HORC, Royal College of Surgeons in Ireland Division of Population Health Sciences, Dublin, Leinster, Ireland

**Could you clarify why your inclusion criteria specify stroke patients aged 19 years and above, while many studies define adults as ≥18 years? You mention that 19 years is the cut-off in PubMed, but since you are also searching Embase and Web of Science, do you think this filter could exclude relevant studies?**

**Response:** Thank you for the feedback and drawing our attention to this.

 As mentioned above to reviewer 1, upon further consideration, we agree that using the PubMed age cut-off is not sufficient. Our review will focus on studies conducted on adults (i.e., aged ≥18 years). We will make the necessary changes to the protocol and in our searches. As suggested by reviewer 1, we will remove the age filter from our search strategy. Exclusion of studies by age will be applied during the screening of the identified articles.

**The decision to restrict to English-language publications may introduce language and/or region bias. Economic studies typically pertain to a local or national setting whereas studies on clinical outcomes may be more or less generic. How do you plan to address or acknowledge this limitation in your review? You apply English-language filter in your search. I would suggest keep all languages. Where possible, you may compare results based on English and non-English publication to point out differences (at least based on abstract information).**

**Response:** We thank you for this feedback.

 The main reason we are excluding studies not published in English is that our review team lacks expertise in language translation. We acknowledge the existence of online software tools that assist in translation, but we believe it is unethical to send other people’s work to third parties for translation. Unfortunately, the research team does not have additional external support and resources to help with translation. We believe that this language restriction is reasonable and accept that we might miss out on cost analyses reported in other languages. However, we also believe that using the English language as a criterion will help in identifying high-quality papers published in international journals rather than local language journals.

**Your focus is on survey-based observational studies. Will you also consider cost data collected via questionnaires in long-term follow-up phases of clinical trials? These might be equally informative when trying to establish long-term costs after achieving a short-term clinical treatment effect. Your protocol excludes experimental and quasi-experimental designs (including randomized and non-randomized controlled trials). It may be worth clarifying whether post-trial follow-up RCTs with survey-based cost data will be included or excluded.**

**Response: **Thank you for your suggestion.

 We agree that post-trial follow-up survey-based studies may be interesting. However, they will not be considered in our study. We decided to exclude these studies from our scoping review as we have identified a research gap in the cost methodologies used in observational studies. We see follow-up studies to RCTs as more suitable for inclusion in another scoping review, which includes RCTs, as they will allow comparison of the settings. We will amend our inclusion and exclusion criteria to specify the exclusion of post-trial follow-up RCTs with survey-based cost data from our scoping review.

**The inclusion of perspective, costing approach, and resource use categories is excellent. Will you also extract details on time horizon (e.g., costs reported over 3 months, 1 year, or longer)? This factor can influence cost estimates and would be valuable to capture.**

**Response:** Thank you for bringing this to our attention.

 We will amend the extraction form to include the time horizon for cost reporting in our data extraction tool.

# Reviewer report: Reviewer response for version 1

**DOI:** 10.21956/hrbopenres.15631.r48935

**Published:** 2025-08-12

**Authors:** John Grosser

**Affiliations:** 1 Universität Bielefeld, Bielefeld, Germany

I believe literature reviews that focus primarily on the methodology of the included studies are an underrated research tool and should be conducted more often. Therefore, I was happy to review this (generally well-written) protocol for a scoping review investigating the cost methodologies of survey-based observational studies in stroke patients. The justification for conducting this review is sufficiently clear from the introduction, and I have mostly minor comments to report.

**Major Comments:**

 1. You say you include only patients aged 19+, rather than 18+, due to PubMeds age categories. I would very strongly advise **not **to use the PubMed age filters, and instead perform a manual screening of the studies resulting from your search string. Predefined age filters may lead to errors and falsely excluded studies. This would also allow you to include studies which include patients who are 18.

 2. Overall, I would strongly recommend **against **using **any **filters in your searches (other than the date, if you decide to keep the date cutoff, see minor comment B). All of these filters run the risk of falsely excluding relevant studies. Instead, I would just use the search string and check for exclusion criteria manually.

 3. You write that the database searches were completed and screening was initiated before the protocol was published. In my view, this is a cause for concern, particularly since this makes it difficult to address major comment 2. In the OSF registry, however, you write that *"Initial searches were conducted to test the search terms. The search phase will properly begin once the review protocol is registered." *This seems contradictory. Have the searches been completed or not? If not, please consider major comments 1 and 2 before completing the searches. If they are already completed, you should check how many studies are filtered out by each database through the application of the filters used, then (re-)screen those particular studies to ensure non are falsely excluded.

**Minor Comments:**

 A. It might be nice to have a table showing the inclusion and exclusion criteria at a glance.

 B. The 20 year cutoff seems somewhat arbitrary. Is there an additional justification you can provide for this choice? Otherwise, I would recommend just screening all articles up to the search date.

 C. The heading structure is somewhat confusing. You have seven topline headings: (1) Introduction, (2) Protocol, (3) Dissemination, (4) Study Status, (5) Tracked and dated amendments, (6) Conclusions and (7) Ethical Considerations. Usually, I would expect a protocol, like any other manuscript, to consist (at minimum) of an introduction, a methods section, a results section, and a discussion section. Please rework the structure or provide a rationale for the existing structure.

 D. You write: '"Studies on either or both male and female biological sex groups will also be considered." This reads somewhat strangely. Will you exclude studies that include intersex patients? Is there a reason to highlight *biological sex groups* at this point in the manuscript? If sex/gender are not particularly relevant to your data extraction and analysis (and it does not seem to be), I would strongly recommend removing this sentence entirely.

Is the study design appropriate for the research question?

Yes

Is the rationale for, and objectives of, the study clearly described?

Yes

Are sufficient details of the methods provided to allow replication by others?

Partly

Are the datasets clearly presented in a useable and accessible format?

Not applicable

Reviewer Expertise:

Relevant areas of expertise: Stroke, Scoping Reviews

I confirm that I have read this submission and believe that I have an appropriate level of expertise to confirm that it is of an acceptable scientific standard, however I have significant reservations, as outlined above.

# Response

**Published:** 2025-10-23

**Authors:** Tumeliwa Mphepo

**Affiliations:** HORC, Royal College of Surgeons in Ireland Division of Population Health Sciences, Dublin, Leinster, Ireland

**Major Comments:**

**You say you include only patients aged 19+, rather than 18+, due to PubMeds age categories. I would very strongly advise not to use the PubMed age filters, and instead perform a manual screening of the studies resulting from your search string. Predefined age filters may lead to errors and falsely excluded studies. This would also allow you to include studies which include patients who are 18.**

**Response:** Thank you for the feedback and drawing our attention to this.

 Upon further consideration, we agree that using the PubMed age cut-off is not sufficient. Our review will focus on studies conducted on adults (i.e., aged ≥18 years). We will make the necessary changes to the protocol and in our searches. As suggested, we will remove the age filter from our search strategy. Exclusion of studies by age will be applied during the screening of the identified articles.

**Overall, I would strongly recommend against using any filters in your searches (other than the date, if you decide to keep the date cutoff, see minor comment B). All of these filters run the risk of falsely excluding relevant studies. Instead, I would just use the search string and check for exclusion criteria manually.**

**Response: **Thank you for this feedback.

 We will take your suggestion on board and run the search with only the date filter. We will still maintain the year-of-publication filter because we believe a 20-year window will yield a more recent and relevant trend in the methods used, which we can apply in future studies. This also relates to restrictions on our time, and we believe 20 years will be sufficient. We have no intentions of documenting a historical development in themes or volume of publications.

**You write that the database searches were completed and screening was initiated before the protocol was published. In my view, this is a cause for concern, particularly since this makes it difficult to address major comment 2. In the OSF registry, however, you write that "Initial searches were conducted to test the search terms. The search phase will properly begin once the review protocol is registered." This seems contradictory. Have the searches been completed or not? If not, please consider major comments 1 and 2 before completing the searches. If they are already completed, you should check how many studies are filtered out by each database through the application of the filters used, then (re-)screen those particular studies to ensure non are falsely excluded.**

**Response:** Thank you for noting this.

 By the time the study protocol was sent for publication, we had only conducted an initial search to test the search terms. No work has begun as the team was waiting for reviewers’ feedback. As suggested, our searches will apply only the date filter. We will remove this statement in the revised protocol.

**Minor Comments:**

**It might be nice to have a table showing the inclusion and exclusion criteria at a glance.**

**Response:** We thank you for suggesting this.

 We will include this table in the revised protocol.

**The 20 year cutoff seems somewhat arbitrary. Is there an additional justification you can provide for this choice? Otherwise, I would recommend just screening all articles up to the search date.**

**Response:** Thank you for this feedback.

 As indicated in response 2 above, we will still maintain the year-of-publication filter because we believe a 20-year window will be a reasonable balance against our time restriction and, at the same time, yield a more recent and relevant trend in the methods used, which we can apply in future studies. This also relates to restrictions on our time, and we believe 20 years will be sufficient. We have no intentions of documenting a historical development in themes or volume of publications.

**The heading structure is somewhat confusing. You have seven topline headings: (1) Introduction, (2) Protocol, (3) Dissemination, (4) Study Status, (5) Tracked and dated amendments, (6) Conclusions and (7) Ethical Considerations. Usually, I would expect a protocol, like any other manuscript, to consist (at minimum) of an introduction, a methods section, a results section, and a discussion section. Please rework the structure or provide a rationale for the existing structure.**

**Response:** We thank you for your comments on this.

 The guidelines for a protocol preparation to be sent to HRB Open specifies that the protocol has to have sections on: authors, title, abstract, keywords, main body, data (and software) availability, reporting guidelines (if applicable), author contributions, competing interests, grant information, acknowledgements (optional), supplementary material, references, figures and tables (if applicable) and images (if applicable). The HRB Open guidelines further state that the main body should include sections on introduction, protocol, and discussion/conclusion. This link provides further details https://hrbopenresearch.org/for-authors/article-guidelines/study-protocols. In our protocol, the sections of dissemination, study status and tracked and dated amendments are part of the protocol section, while ethical consideration is part of the conclusion section.

You write: '"Studies on either or both male and female biological sex groups will also be considered." This reads somewhat strangely. Will you exclude studies that include intersex patients? Is there a reason to highlight *biological sex groups* at this point in the manuscript? If sex/gender are not particularly relevant to your data extraction and analysis (and it does not seem to be), I would strongly recommend removing this sentence entirely.

**Response:** Thank you for this feedback.

 We understand the confusion this statement brings. The intention was to emphasise that studies of one sex alone would be included. This statement will be removed in the revised version of the protocol.
